# Pulmonary embolism presenting with itinerant chest pain and migratory pleural effusion: A case report

**DOI:** 10.1097/MD.0000000000010944

**Published:** 2018-06-01

**Authors:** Wei Li, Chen Chen, Mo Chen, Tong Xin, Peng Gao

**Affiliations:** a Department of Respiratory and Critical Care Medicine, The Second Hospital of Jilin University, Changchun, Jilin; b Department of Respiratory, The Fourth Hospital of Daqing City, DaQing, Helongjiang, China.

**Keywords:** chest pain, computed tomographic pulmonary angiography, D-dimer, pulmonary embolism, pleural effusion

## Abstract

**Introduction:**

Pulmonary embolism (PE) presents with complex clinical manifestations ranging from asymptomatic to chest pain, hemoptysis, syncope, shock, or sudden death. To the authors’ knowledge, itinerant chest pain has not been reported as sign or symptom of PE.

**Case presentation:**

A 41-year-old woman presenting with left chest pain, no hemoptysis, or breathing difficulties. The chest pain was more severe on deep inspiration. Chest computed tomography (CT) and ultrasound imaging showed left pleural effusion. After antibiotic treatment, the left chest pain was alleviated, but a similar pain appeared in the right chest. Electrocardiogram, blood gas analysis, echocardiography, and D-dimer levels were unremarkable. Chest CT showed right pleural effusion. A CT pulmonary angiography (CTPA) unexpectedly revealed a PE in the right pulmonary artery. The patient was administered anticoagulant therapy and made a complete recovery.

**Conclusions:**

The use of CTPA to investigate the possible presence of PE in patients with unexplained migratory pleural effusion complaining of itinerant chest pain is important. Lessons should be learned from the early use of CTPA to investigate the possible presence of PE in patients.

## 1 Introduction

Pulmonary embolism (PE) is the blockage of one or more pulmonary arteries by emboli.^[1]^ Clinical signs of PE are nonspecific, ranging from occult to hemodynamic instability and even sudden death. Common symptoms include unexplained dyspnea and shortness of breath, chest pain, syncope, irritability, panic with a sense of impending doom, hemoptysis, cough, and palpitations.^[2]^ To the authors’ knowledge, itinerant chest pain and migratory pleural effusion are not common symptoms of PE. In patients with atypical symptoms, diagnosis and treatment of PE may be delayed, which can have a fatal outcome.^[3]^

Here, we present a case of PE characterized by itinerant chest pain and accompanied by migratory pleural effusion. Findings should alert physicians that vigilance is needed to identify patients with atypical symptoms of PE or occult PE.

## 2 Case presentation

**Figure 1:**
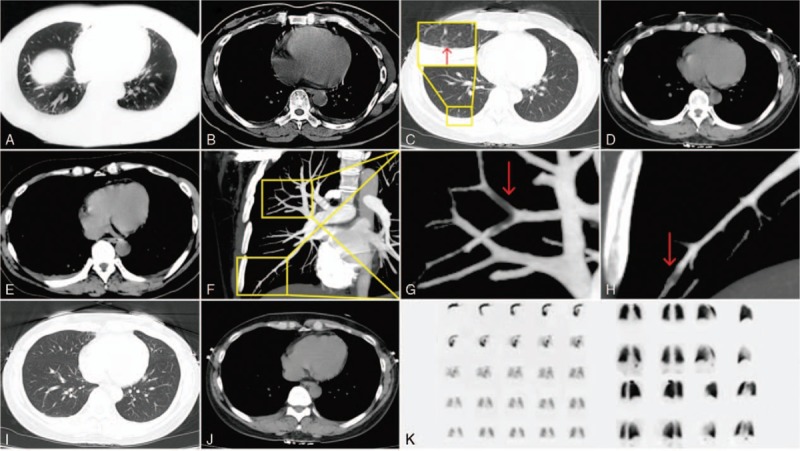
Chest CT, CTA, and lung perfusion images. A, B, Chest CT scan 3 days before admission showed a small left pleural effusion. C,D Chest CT scan 1 day after admission showed scattered small ground-grass opacities in the bilateral lung field, but no pleural effusion in the left lung. E, Chest CT scan 3 days after admission showed right pleural effusion. F–H, CT pulmonary angiography (CTPA) 5 days after admission showed an embolism in the right pulmonary artery. I–K, Chest CT and lung perfusion scan 3 months after discharge were normal.

**Figure 2:**
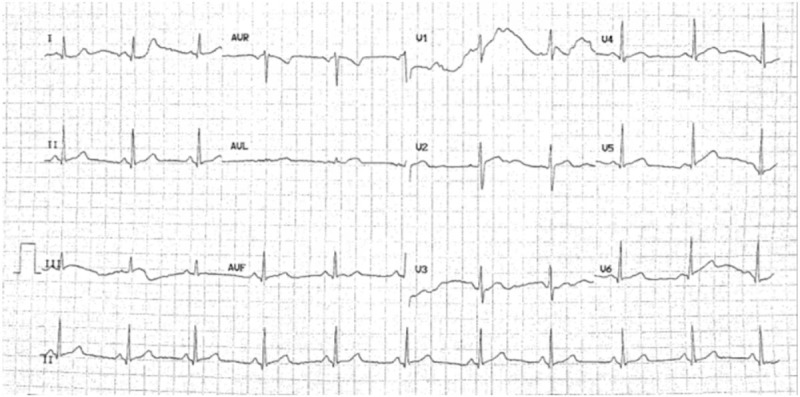
Sinus rhythm and ST-T wave changes indicated myocardial ischemia.

A 41-year-old woman presented to another institution with persistent left chest pain for 8 days, but no incident cause or other complaints. The chest pain was more severe when the patient took a deep breath. The patient had no history of recent surgery or deep venous thrombosis, she had never taken oral contraceptives, and she denied drinking alcohol and smoking cigarettes. A chest computed tomography (CT) scan showed scattered small ground-grass opacities in the bilateral lung field and a well-defined dense shadow in the left lung (Fig. 1 A,B). Chest ultrasound confirmed left pleural effusion. The patient was diagnosed with double pneumonia and left pleural effusion. The patient received antibiotics for 8 days, which slightly alleviated the left chest pain. The patient was transferred to our hospital for further diagnosis and treatment. On admission her clinical parameters were body temperature 36.5°C, pulse 71 beats/min, respiratory rate 15 breaths/min, blood pressure 118/85 mm Hg, and oxygen saturation when breathing room air 98%. Physical examination was unremarkable. White blood cell count, liver function, kidney function, myocardial markers, and brain natriuretic peptide values were normal, and D-dimer level was 0.02 mg/L. A repeat chest CT scan on the first day after admission showed scattered small ground-grass opacities in the bilateral lung field, but no pleural effusion in the left lung (Fig. 1C,D). ECG revealed sinus rhythm and ST-T wave changes, and myocardial ischemia was suspected (Fig. 2). Echocardiography showed that ejection fraction was 77%, the right ventricle end-diastolic diameter was 23 mm, tricuspid valve regurgitation, and a valve area of approximately 2.0 cm^2^. Abdominal ultrasound showed no abnormalities of the liver, gallbladder, pancreas, spleen, or kidney. Double pneumonia was suspected, and the patient was prescribed another course of antibiotics. Two days later, the patient's left chest pain was alleviated; however, a similar but more severe pain appeared in the right chest. Further clinical assessments showed the patient's D-dimer level was 0.08 mg/L, and chest CT scan revealed right pleural effusion had emerged (Fig. 1E). After discussion amongst the clinical team, a computed tomographic pulmonary angiography (CTPA) was performed. Findings were consistent with a PE in the right pulmonary artery (Fig. 1F–H), and a small amount of pleural effusion was seen on the right. No filling defects were seen in the bilateral lower limb veins.

The patient was treated according to the guidelines for the diagnosis and treatment of PE.^[4]^ Low-molecular-weight heparin calcium injection 4100 IU was administered twice daily by subcutaneous injection. Chest pain was fully alleviated after 6 days, and oral anticoagulant rivaroxaban was given after discharge. Three months later, a lung perfusion scan showed the PE in the right pulmonary artery had significantly improved, and ultrasound showed no evidence of pleural effusion (Fig. 1I–K).

## 3 Discussion

PE is a general term for a group of diseases or clinical syndromes, including pulmonary thromboembolism, fat embolism syndrome, amniotic fluid embolism, and air embolism, in which emboli block one or more pulmonary arteries.^[5,6]^ Clinical manifestations of PE are complex and diverse, and the rates of clinical misdiagnosis and missed diagnosis are high. Globally, PE is a cause of substantial acute and chronic morbidity. PE is also associated with mortality, with >30% of PE patients dying during the initial 30 days after diagnosis. As correct management of PE can reduce the mortality rate to <10%,^[7]^ timely and accurate diagnosis and treatment of PE are essential.

In the current study, the patient initially presented with left chest pain; she was diagnosed with double lung pneumonia and left pleural effusion.^[8]^ Pleural effusion may be caused by heart failure, pneumonia, cancer, and tuberculosis. In the current study, based on the patient's clinical manifestations, the chest pain was initially thought to result from parapneumonic effusion. However, administration of anti-inflammatory treatment only slightly reduced the left chest pain. New pain appeared in the right chest, which did not support a diagnosis of parapneumonic effusion; therefore, we considered occult PE.

Evidence suggests that pleural effusions occur in 19% to 61% of patients with PE.^[7]^ Pleural effusions secondary to PE are usually exudates. PE causes pleural effusion by increasing pulmonary capillary permeability, such that pulmonary interstitial fluid moves from the lung to the pleural space by crossing the visceral pleura. The permeability of pulmonary capillaries may be increased by the release of inflammatory mediators from clots that are rich in platelets. These inflammatory mediators include vascular endothelial growth factor (VEGF), which is among the most effective vascular permeabilizing factors known. Large amounts of VEGF are contained within platelets. Permeability may also be increased by ischemia of the pulmonary capillaries distal to the embolus; however, this effect is likely to be minimal as the bronchial circulation provides blood to these capillaries.^[9]^

In the current study, we describe a patient with PE and migratory pleural effusion in which the left pleural effusion migrated to the right following antibiotic treatment. Studies in patients with unilateral pleural effusion show no relationship between the side of the pleural effusion and the side of the PE.^[10]^ Porcel et al analyzed 230 patients with PE and found pleural effusions in 93 patients. Of these, 61 patients had a unilateral PE, 38 patients had an ipsilateral pleural effusion, and 7 patients had a contralateral pleural effusion; in 16 patients, pleural effusion was bilateral. The authors concluded that the side of the pleural effusion did not correlate with the side of the PE, but they cautioned that conventional CTA may have low sensitivity for the detection of emboli in subsegmental pulmonary arteries; therefore, a contralateral pleural effusion may be an artifact. Indeed, emboli are often found in many segments or lobes of the lung in autopsy studies.^[11]^

Pleural effusions often develop in patients with right heart failure.^[12]^ Most PE is accompanied by pulmonary hypertension, and thus right ventricular dysfunction. Evidence suggests that patients with pleural effusions and right heart failure have significantly elevated mean right atrial pressure, which leads to an increase in central and systemic venous pressures and the formation of pleural fluid, as parietal pleural lymphatic drainage is mechanically impeded, or because a transudate forms in the pleural space due to increased venous and hydrostatic pressures in the veins of the bronchae and chest wall.^[13]^ In the current study, we propose that a previously undetected PE that underwent subsequent dissolution caused the pleural effusion. In support of this, Agarwal et al^[14]^ indicated that pleural effusions associated with PE reach a maximum size by day 3, and further growth of the effusion or the appearance of a contralateral effusion is indicative of a recurrent embolism.

Kiris et al^[15]^ reported acute PE patients with pleural effusion had a significantly higher incidence of all-cause and long-term total mortality than those without pleural effusion. Unfortunately, if the amount of pleural fluid is small, pleural aspiration cannot be performed in these patients.

In the current study, the patient's D-dimer level was not remarkable. Generally, there is a dose-response relation between D-dimer level and risk for a PE. However, a PE diagnosis should not be made based on D-dimer levels alone as the test lacks specificity. The simultaneous occurrence of acute thrombosis, coagulation, and activation of fibrinolysis can elevate plasma D-dimer levels. If D-dimer levels are within the normal range, a diagnosis of PE can almost be excluded if the patient's clinical probability score for a PE is low. In patients with high D-dimer levels and a high clinical probability of PE, further tests should be used to make a definitive diagnosis.^[16,17]^ In clinical practice, PE can be easily missed or misdiagnosed based on a normal D-dimer level. We recommend the use of CTPA to investigate the possible presence of occult PE in patients with unexplained pleural effusion and normal plasma D-dimer levels.

In the current study, as the patient had no risk factors for PE, thrombophilia may not be excluded. Thrombophilia (hypercoagulability; prothrombotic state) results from a genetic or acquired deficiency in an anticoagulant protein, clotting factor, or fibrinolytic protein.^[18]^ Patients with thrombophilia have a higher incidence of deep venous thrombosis and PE, collectively known as venous thromboembolism. Inherited thrombophilia is usually due to mutations in the coagulation factor V (factor V Leiden) and prothrombin (prothrombin G20210A) genes. Acquired thrombophilia may be associated with old age, obesity, inflammatory bowel disease, malignancy, smoking, a history of thrombosis, prolonged alcohol use, oral contraceptives, hormone replacement therapy, trauma, pregnancy and birth, and antiphospholipid syndrome. Nephrotic syndrome, myeloproliferative disorders, paroxysmal nocturnal hemoglobinuria, stroke, heart failure, myocardial infarction, and diabetes mellitus also cause elevated levels of coagulation factors and decreased levels of anticoagulants.^[19]^ We recommend that genetic testing should be considered in patients with a diagnosis of PE but an unremarkable medical history and no risk factors.

## 4 Conclusion

Based on the findings from the current study, we recommend that PE should be considered in patients complaining of itinerant chest pain, especially pleuritic chest pain, with unexplained migratory pleural effusion.

## Author contributions

WL and LW carried out the data collection, literature review and drafting of the manuscript. MC contributed to the drafting of the manuscript and aided in the literature review. TX participated in the data collection and the drafting of the manuscript. PG help to draft the manuscript and revised the final version of the manuscript. All authors read and approved the final manuscript.

**Data curation:** Tong Xin.

**Investigation:** Chen Chen.

**Resources:** Mo Chen.

**Supervision:** Peng Gao.

**Writing – original draft:** Wei Li.
